# Prophylactic and Therapeutic Use of Strontium Ranelate Reduces the Progression of Experimental Osteoarthritis

**DOI:** 10.3389/fphar.2018.00975

**Published:** 2018-09-19

**Authors:** Thiago A. Rodrigues, Abner de Oliveira Freire, Heetor C. O. Carvalho, Gyl E. B. Silva, José W. Vasconcelos, Rosane N. M. Guerra, Maria do Socorro de Sousa Cartágenes, João B. S. Garcia

**Affiliations:** Centro de Ciências Biológicas e da Saúde, Universidade Federal do Maranhão, São Luís, Brazil

**Keywords:** strontium ranelate, osteoarthritis, pain, treatment, inflammation

## Abstract

**Introduction:** Strontium ranelate (SrRan) has the potential to interfere in the progression of osteoarthritis (OA), multifactorial disease associated with mechanical problems and articular inflammatory changes.

**Objectives:** This study aimed to test the effects of prophylactic and therapeutic use of SrRan on clinical parameters of pain, the inflammatory process, and degradation of the articular cartilage.

**Methods:** This was an experimental study, using a model of knee OA induced by intra-articular injection of monoiodoacetate. Thirty Wistar rats were divided into five groups and treated as indicated: control, without intervention; prophylactic, received SrRan at a daily oral dose of 250 mg/kg for 28 days before OA induction; SrRan treatments, administered 250 or 500 mg/kg/day for 28 days after the induction; and model control, received saline solution after the induction. Behavioral tests (joint incapacity, mechanical hyperalgesia, tactile sensitivity, and forced ambulation), histological evaluation of articular cartilage, and determination of inflammatory cytokines in the synovial fluid (interleukin [IL]-6, IL-10, tumor necrosis factor [TNF]-α, and interferon [INF]-γ) were performed.

**Results:** Both prophylactic and therapeutic treatments improved the articular discomfort. A prophylactic dose of 500 mg/kg/day also improved mechanical hyperalgesia and the same dose was beneficial on tactile sensitivity. SrRan did not improve ambulation. Levels of IL-6, IL-10, TNF-α, and IFN-γ in SrRan-treated groups with OA were not significantly different compared with those in the normal control animals. The histopathological evaluation showed less articular damage in the SrRan-treated and control groups compared to the saline-treated group.

**Conclusion:** The prophylactic and therapeutic administration of SrRan was associated with improved behavioral patterns of pain, especially joint discomfort. SrRan administration mitigated histological changes in the articular cartilage and reduced the inflammatory process, which beneficially reduced the progression of OA in the experimental model studied.

## Introduction

A greater understanding of the pathophysiology of osteoarthritis (OA) has gradually transformed the classic concept of articular degenerative disease, emphasizing mechanical and inflammatory phenomena in its pathogenesis and progression (Berenbaum, 2013; Felson, 2013). OA is characterized by cellular stress and degradation of the extracellular matrix, caused by macro- and micro-injuries that activate maladaptive repair responses (Kraus et al., 2015).

The emergence of OA can be related to a response to mechanical insults, leading to an abnormal increase of forces in certain areas of the joints. Congenital or acquired anatomical alterations and joint overload are commonly associated with both increased static and dynamic local stress, generating bad alignment and related injuries. Consequently, the articular tissues lose their ability to adequately support the loads imposed on them (Felson, 2013).

Besides cartilage, other joint components including synovial fluid, ligaments, and adjacent bone tissue play relevant roles in the pathophysiology of OA. The importance of the subchondral bone in the pathogenesis and development of OA has been recently highlighted, suggesting that it interferes with chondrocyte metabolism (Funck-Brentano and Cohen-Solal, 2015).

The most accepted proposed mechanism implicates vascular changes in the subchondral bone in changes in the cartilaginous matrix, through the reduction of intra-osseous perfusion and consequent hypoxia (Aaron et al., 2017). Furthermore, the consequent physical-chemical disturbances are capable of generating responses by osteoblasts with the production of a modified profile of cytokines involved in the degeneration of the articular cartilage and catabolic activity of the chondrocytes (Aaron et al., 2017). The chondrocytes and synovial cells produce increased levels of inflammatory cytokines such as interleukin (IL)-1β and tumor necrosis factor (TNF)-α, which decrease the synthesis of collagen and increase catabolic mediators, such as metalloproteinases (MMPs), and other inflammatory mediators, such as IL-8, IL-6, prostaglandin E2 (PGE2), and nitric oxide (NO) (Berenbaum, 2013).

There has been a constant search for substances that can be combined with conventional therapy for OA. Currently, conventional therapy consists of a combination of non-pharmacological measures such as aerobic exercises, weight loss, and joint protection techniques, as well as symptomatic pharmacological treatments including anti-inflammatory non-steroidal analgesics and corticosteroids or local intra-articular lubricants until, eventually, surgical intervention is required (Lafeber and Van Laar, 2013; Gelber, 2015).

Strontium ranelate (SrRan), an antiresorptive agent and bone proforming agent already proven effective in patients with severe osteoporosis, has been the subject of clinical and experimental studies in OA because of a probable effect on both bone turnover and inflammation associated with this disease (Reginster et al., 2005; Reginster, 2014; Tenti et al., 2014; Rodrigues et al., 2017).

The exact mechanism of action of SrRan is not fully understood. However, regulation of bone cell differentiation, stimulation of osteoblast proliferation, and inhibition of osteoclast formation with probable apoptosis of “mature” cells, in addition to the activation of calcium-sensitive receptors have been considered as possible mediators of the pharmacological properties of this medication (Brennan et al., 2009; Fonseca and Brandi, 2010; Lems and Geusens, 2014). The inhibition of osteoclastic activity by SrRan has been demonstrated to be related to the reduction in MMP synthesis and modulation of the osteoprotegerin-receptor activator of nuclear factor kappa-B ligand (RANKL) pathway (Tat et al., 2011).

In line with the current trend of investigating drugs that can control the inflammatory component of OA, pre-clinical studies with SrRan have been performed to test its possible effect in this setting. However, results showing the probable antinociceptive effects of SrRan have conflicted with those of alterations in levels of inflammatory mediators such as TNF-α and IL1-β (Nunes et al., 2015; Alves et al., 2017; Mierzwa et al., 2017).

There is accumulating evidence of the action of SrRan on both articular cartilage and subchondral bone, but few studies have investigated the actions of this drug on OA. In addition, considering that inflammation is one of the main mediators of OA progression, this study aimed to examine the effects of SrRan in an experimental rat model of OA, using clinical and histological evaluations as well as the assessment of inflammatory cytokines. We also aimed to use this preclinical model to compare the potential protective effects of SrRan following prophylactic use with its eventual therapeutic action on articular cartilage in OA.

## Materials and Methods

The study was conducted at the Experimental Laboratory for Study of Pain (LEED), after approval by the Ethics Committee on Animal Use of the Federal University of Maranhão - Brazil (CEUA-UFMA number 23115.012456/2016-4).

### Animals

Thirty male, approximately 60-day-old Wistar rats, *Rattus norvegicus* species (albinus variety) were used in the study. The animals were obtained from the Central Animal Facility of the Universidade Federal do Maranhão. They remained in the bioterium of the LEED and were fed a standard ration and water *ad libitum* and maintained under controlled conditions of light and temperature.

### Experimental Protocol

**FIGURE 1:**
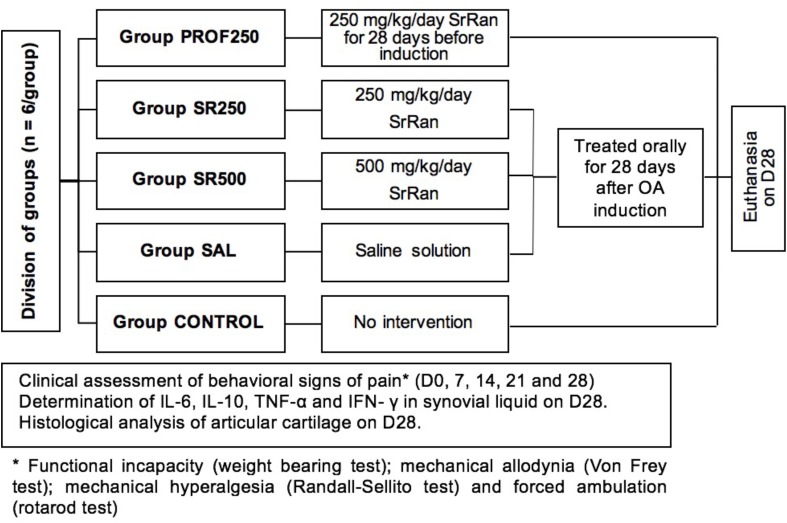
Experimental protocol.

The animals were divided into five groups (PROF250, SR250, SR500, SAL, and CONTROL) of six rats each. For the PROF250 (“prophylactic”) group, from 4 weeks before the induction of OA with sodium monoiodoacetate (MIA), each rat received SrRan at a dose of 250 mg/kg by gavage once daily in the morning, 2 h before the subsequent feeding. The SR250 and SR500 (”treated”) groups received SrRan at doses of 250 and 500 mg/kg, respectively, by gavage once daily in the morning, 2 h before the subsequent feeding for 28 days. The SAL group received saline solution (0.9% sodium chloride) by gavage for 21 days from day 7 after the induction of OA. The day of OA induction in groups PROF250, SR250, and SR500 and saline administration was considered D0. Further, OA was not induced in the CONTROL group, which was administered only saline solution by gavage. Throughout the experiment, all groups were evaluated periodically for joint incapacity, mechanical allodynia, mechanical hyperalgesia, and motor activity by forced ambulation on D0, D7, D14, D21, and D28 (**Figure 1**). On D28, after completing the clinical evaluations, synovial fluid was collected from the affected joint of each rat for the laboratory analysis of cytokines, the animals were euthanized with a lethal intraperitoneal injection of sodium thiopental (100 mg/kg), and then, the articular cartilage was harvested for histopathological analysis.

### Sodium MIA-Induced OA Model

For the induction of OA, the animals were anesthetized with intraperitoneal injections of 40 mg sodium thiopental. After certifying the anesthetic plane, a trichotomy was performed in the right knee and, subsequently, a topical solution of 10% iodopovidone was applied for local asepsis. An articular lesion was induced by a single intra-articular injection of 2 mg sodium MIA (diluted in a maximum volume of 25 μL) into the right knee through the patellar ligament (Fernihough et al., 2004; Silva et al., 2008).

### Clinical Evaluations

#### Weight-Bearing Test/Weight Distribution Test on Hind Legs

The animals were placed in a glass bowl angled and positioned so that each hind leg laid on different platforms. The weight exerted on each back paw (measured in grams) was evaluated for 5 s. The final measurement of weight distribution was the mean of three measurements. Changes in the weight distribution on the paws were calculated as follows:

$$\text{Weight distribution }(\%)=\frac{\text{APW}}{\text{APW}+\text{CPW}}\times 100$$

where APW was affected paw weight and CPW was contralateral paw weight (Schött et al., 1994).

#### Quantification of Mechanical Allodynia (*Von Frey Test*)

The evaluation of mechanical allodynia was performed using an electronic device (Model 1601C, Life Science, San Francisco, CA, United States), which consisted of a pressure transducer connected to a digital force counter expressed in grams (g) and calibrated to record a maximum force of 150 g. The animals were placed in individual transparent acrylic boxes on raised platforms to allow access to the lower part of their bodies. The holes in the platforms provided access to the transducer tip, allowing its contact with the animals’ paws. The response of the paw withdrawal frequency to the filament stimulus was measured in five applications, lasting 1 s each, always performed by the same evaluator, and the final result was the mean of all measurements (Möller et al., 1998).

#### Mechanical Hyperalgesia (*Randall Selitto Test*)

Mechanical hyperalgesia was assessed by evaluating the nociceptive threshold paw withdrawal following the application of mechanical pressure using an analgesiometer (model IITC, Life Science). A wedge-shaped device (area, 1.75 mm^2^) was applied to the dorsal surface of the hind paws with increasing linear pressure until the animal responded by withdrawing the paw. Three measurements were performed in the ipsilateral and contralateral paws. A cut-off threshold pressure of 250 g was programmed to prevent tissue damage.

The paw withdrawal reflex was considered to represent the hypernociceptive threshold. The nociceptive paw withdrawal threshold (NPWT) was recorded in grams and defined as the percentage pressure required to provoke a withdrawal of the ipsilateral affected paw, and was calculated as follows:

$$\text{NPWT }(\%)=\frac{\text{NAPWT}}{\text{NAPWT}+\text{NCPWT}}\times 100$$

where NPWT was nociceptive paw withdrawal threshold, NAPWT was nociceptive affected paw-withdrawal threshold and NCPWT was nociceptive contralateral paw- withdrawal threshold (Randall and Selitto, 1957; Nogueira et al., 2012).

#### Evaluation of Motor Activity/Forced Ambulation (*Rotarod Test*)

The animals were placed on a swivel bar (model IITC, Life Science) at a speed of 16 rpm for a period of 300 s. The use of the affected limb was assessed by forced ambulation. The use of the paw was graded using a numerical scale ranging from 5 to 1, where: 5 = normal use of the paw, 4 = mild claudication, 3 = severe claudication, 2 = intermittent disuse of affected paw, and 1 = complete disuse of affected paw (Monville et al., 2006).

### Laboratory Analysis of Cytokines

Laboratory analysis of the synovial fluid to quantify IL-6, IL-10, TNF-α, interferon (IFN)-γ was performed using an enzyme-linked immunosorbent assay (ELISA, R&D Systems^®^, Minneapolis, MN, United States).

The synovial fluid samples were obtained on D28 by washing out the affected knee joint twice with 200 μL phosphate-buffered solution (0.15 M, pH 7.4) containing 37.2 mg ethylenediaminetetraacetic acid (EDTA, 0.01 M).

### Histopathological Analysis of Articular Cartilage

On D28, the articular cartilage and subchondral bone of the knee of each animal were removed after euthanasia. The excised components were embedded in paraffin blocks, cut into 5 μm sections, and the proteoglycans of the organic cartilage matrix were specifically stained using 0.5% safranin-O.

The histopathological evaluation was performed according to the guidelines of the Osteoarthritis Research Society International (OARSI). The slides were analyzed blindly by two pathologists, who graded them on a scale of 0–6, according to the severity of the articular cartilage lesion. The classification considered the most severe lesion observed on the slide regardless of the extent of the lesion. Grade 0 indicated morphologically intact cartilage, grade 1 indicated an intact surface with possible focal lesions or abrasion, grade 2 showed discontinuity in the articular surface, grade 3 showed vertical fissures, grade 4 presented erosion, grade 5 exhibited denudation with sclerotic bone or fibrocartilaginous tissue repair or both, and grade 6 showed remodeling and bone deformation with changes in the contour of the articular surface (Pritzker et al., 2016).

### Statistical Analysis

The means of different experimental groups were compared using the Student’s *t*-test or a univariate (one-way) analysis of variance (ANOVA), followed by the Bonferroni test for multiple comparisons. A *p* < 0.05 was considered statistically significant, and the data obtained were analyzed using the GraphPad Prism 7.0^®^ software for Windows^®^ (CA, United States).

## Results

### Assessment of Joint Incapacity Using (*Weight Bearing Test*)

**FIGURE 2:**
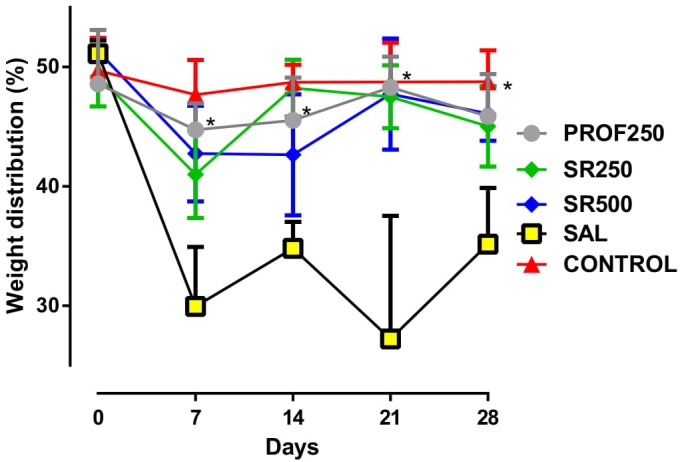
Evaluation of joint disability using weight bearing test. Groups: PROF250, prophylactically administered strontium ranelate (SrRan) 250 mg/kg 4 weeks before osteoarthritis (OA) induction; SR250 and SR500, OA-induced and treated with SrRan 250 and 500 mg/kg, respectively for 4 weeks after induction; SAL, OA-induced and receiving only saline; CONTROL, untreated and not OA-induced. Results are means ± standard deviation (SD). ^∗^*p* < 0.05 comparing groups PROF250, SR250, and SR500 with SAL, using one-way analysis of variance (ANOVA) followed by the Bonferroni test. *X*-axis = Days; *Y*-axis = Weight distribution of paws (%).

Comparisons on D7, D14, D21, and D28 between the prophylactic group (the group pre-treated with SrRan before OA induction - PROF250), and the CONTROL (healthy animals) showed that pre-treated animals may have experienced benefit from using SrRan, as they approached the healthy standard, with greater balance in weight distribution. A similar trend was observed when the SR250 and SR500 groups were compared with the CONTROL: on D7, D14, D21, and D28, the treated animals approached the normal status of the CONTROL group. The results of the PROF250, SR250, and SR500 groups were statistically different from those of the SAL group (animals with OA, receiving saline), demonstrating that the prophylactic and therapeutic use of SrRan at these doses were effective in improving the weight distribution of paws (**Figure 2**).

### Quantification of Mechanical Allodynia (*Von Frey Test*)

**FIGURE 3:**
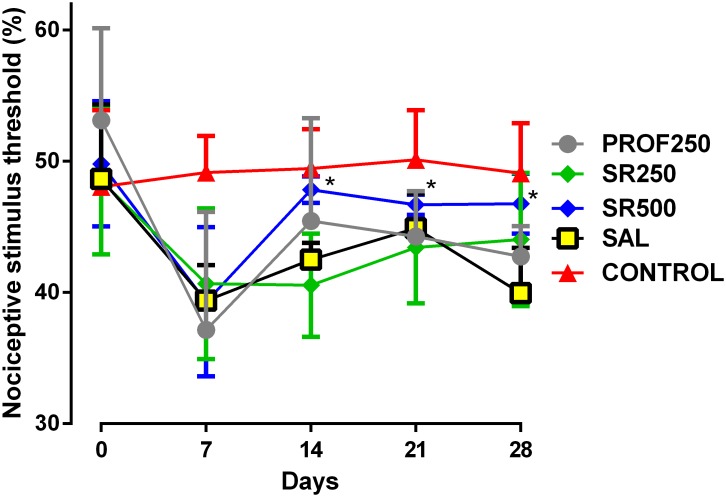
Evaluation of tactile sensitivity using *Von Frey test.* Groups: PROF250, prophylactically administered SrRan 250 mg/kg 4 weeks before OA induction; SR250 and SR500, OA-induced and treated with SrRan 250 and 500 mg/kg, respectively for 4 weeks after induction; SAL, OA-induced and receiving only saline; CONTROL, untreated and not OA-induced. Results are means ± SD; ^∗^*p* < 0.05 from D14 onward, comparing SR500 and SAL groups using one-way analysis of variance (ANOVA) followed by the Bonferroni test. *X*-axis = Days; *Y*-axis = nociceptive stimulus threshold (%).

The PROF250 group did not show any improvement in spontaneous pain behaviors compared to the SAL group. A similar trend was observed when the SR250 and SAL groups were compared, and no beneficial effect was observed in the analysis. A beneficial effect was observed in animals administered 500 mg/kg SrRan from D14 compared to the SAL group (**Figure 3**).

### Mechanical Hyperalgesia (*Randall Selitto Test*)

**FIGURE 4:**
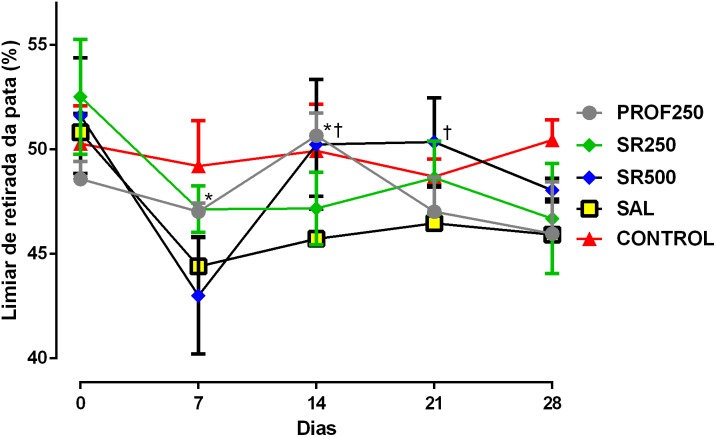
Evaluation of mechanical hyperalgesia using the Randall Selitto test. Groups: PROF250, prophylactically administered SrRan 250 mg/kg 4 weeks before OA induction; SR250 and SR500, OA-induced and treated with SrRan 250 and 500 mg/kg, respectively for 4 weeks after induction; SAL, OA-induced and receiving only saline; CONTROL, untreated and not OA-induced. Results are means ± SD; ^∗^*p* < 0.05 between groups PROF250 and SAL on D7, and D14, using one-way ANOVA followed by the Bonferroni test. ^†^*p* < 0.05 at D14 and D21, in the comparison between SR500 and SAL groups, using *one-way* ANOVA followed by the Bonferroni test. *X*-axis = Days; *Y*-axis = Paw withdrawal threshold (%).

Prophylactic use of SrRan in the PROF250 group led the animals to exhibit responses that were different to those of the SAL group on D7 and D14; such effects did not persist up to D21 and D28. No beneficial effect was observed on mechanical hyperalgesia with 250 mg/kg SrRan compared to the untreated SAL group. At a dose of 500 mg/kg SrRan, isolated improvement in mechanical hyperalgesia was found only on D14 and D21 (**Figure 4**). Thus, there was an inconsistent effect on mechanical hyperalgesia with the use of different doses of SrRan.

### Assessment of Motor Activity/Forced Ambulation (*Rotarod Test*)

**FIGURE 5:**
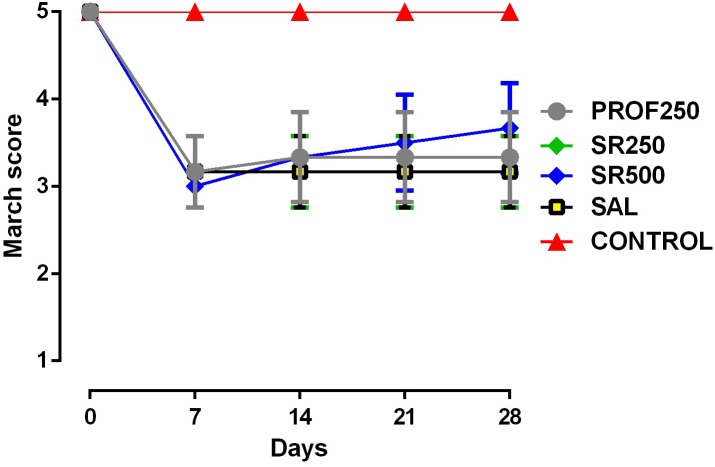
Evaluation of motor activity/forced ambulation using the rotarod test. Groups: PROF250, prophylactically administered SrRan 250 mg/kg 4 weeks before OA induction; SR250 and SR500, OA-induced and treated with SrRan 250 and 500 mg/kg, respectively for 4 weeks after induction; SAL, OA-induced and receiving only saline; CONTROL, untreated and not OA-induced. Results are means ± SD.

No improvement of motor function was observed with prophylactic use and treatment with 250 and 500 mg/kg SrRan compared with the SAL group, with no proven benefit of using SrRan by this specific test (**Figure 5**).

### Laboratory Analysis of Cytokines

The determination of cytokines in the synovial fluid samples collected at the end of the procedure is shown in **Table 1**. The levels of IL-6, IL-10, TNF-α, and IFN-γ in the SrRan-treated PROF250, SR250, and SR500 groups were close to those of the normal CONTROL group. However, a similar trend was not observed between the SAL and CONTROL groups, which showed significantly different values. It was also noted the lack of significant differences among the prophylactic, 250 and 500 mg/kg/day SrRan-treated groups.

**Table 1:** Determination of cytokine levels in synovial fluid using enzyme-linked immunosorbent assay (ELISA).

Groups	TNF-α	IL-6	IFN-γ	IL-10
PROF250	378.2 ± 33.29	95.3 ± 13.8	1377 ± 110.2	638.8 ± 87.5
	(*p* = 0.78)	(*p* = 0.81)	(*p* = 0.82)	(*p* = 0.9)
SR250	392.2 ± 43.89	133.7 ± 14.7	1558 ± 101.5	632.3 ± 87.5
	(*p* = 0.95)	(*p* = 0.54)	(*p* = 0.11)	(*p* = 0.9)
SR500	411.5 ± 25.26	99.4 ± 12.8	1173 ± 137.7	476.2 ± 83.8
	(*p* = 0.99)	(*p* = 0.92)	(*p* = 0.45)	(*p* = 0.35)
SAL	569.9 ± 41.1	173.4 ± 14.6	1891 ± 139.2	303.4 ± 92.8
	(*p* = 0.01)	(*p* < 0.01)	(*p* < 0.01)	(*p* = 0.01)
CONTROL	416.1 ± 33.29	110.5 ± 15.7	1311 ± 115.9	634.9 ± 112.0

*Groups: PROF250, prophylactically administered strontium ranelate (SrRan) 250 mg/kg 4 weeks before osteoarthritis (OA) induction; SR250 and SR500, OA-induced and treated with SrRan 250 and 500 mg/kg, respectively for 4 weeks after induction; SAL, OA-induced and receiving only saline; CONTROL, untreated and not OA-induced. Results are means ± standard deviation (SD); p-values express comparison between OA-induced groups and CONTROL. TNF-α, tumor necrosis factor-α; IL, interleukin; interferon-γ, IFN-γ.*

### Histopathological Analysis of Articular Cartilage

**FIGURE 6:**
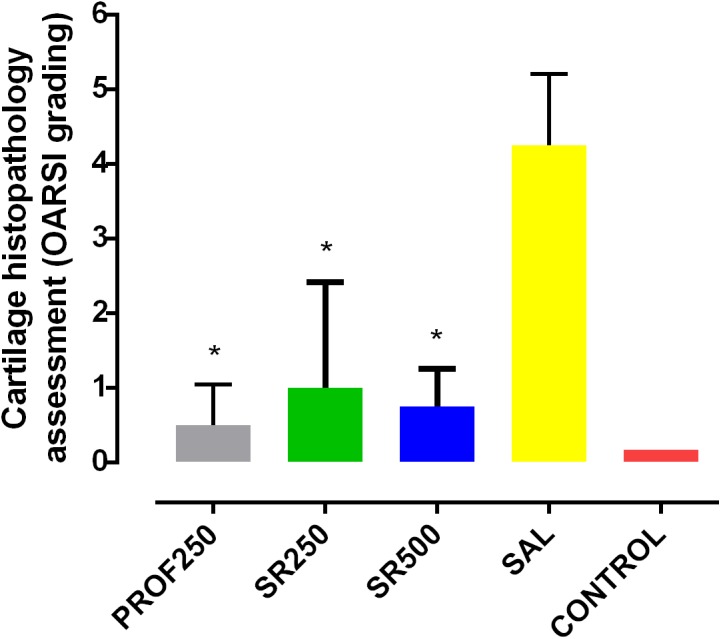
Histopathologic assessment of cartilage classified using the Osteoarthritis Research Society International (OARSI) scoring system. Groups: PROF250, prophylactically administered SrRan 250 mg/kg 4 weeks before OA induction; SR250 and SR500, OA-induced and treated with SrRan 250 and 500 mg/kg, respectively for 4 weeks after induction; SAL, OA-induced and receiving only saline; CONTROL, untreated and not OA-induced. Results are means ± SD; ^∗^*p* < 0.05, comparing PROF250, SR250, and SR500 and SAL groups, using one-way analysis of variance (ANOVA), followed by Bonferroni test. *Y*-axis: Histological assessment of cartilage (OARSI Classification).

**FIGURE 7:**
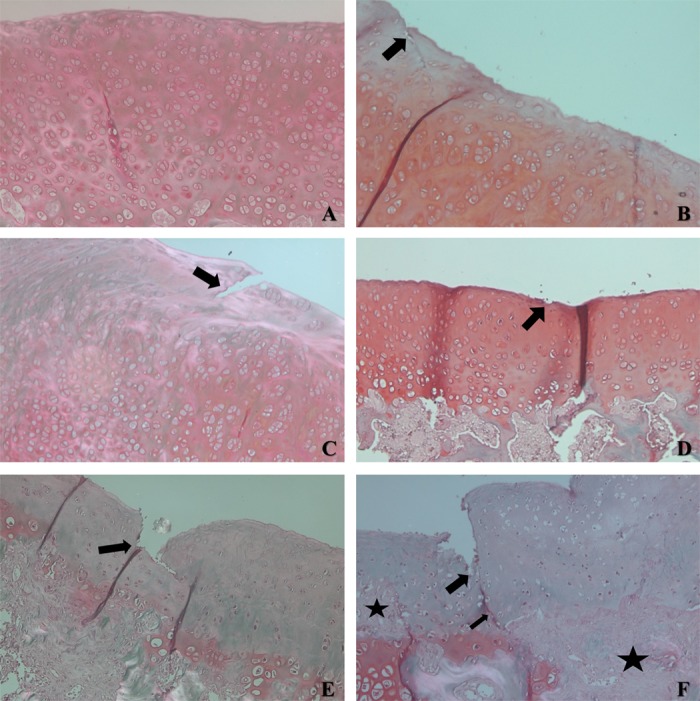
Sections of articular cartilage with different grades of degenerative changes in OA classified using OARSI scoring system. **(A)** Example of the CONTROL group - Grade 0 - normal cartilage. **(B)** Example of PROF250 group - grade 1, indicates intact surface with slight discontinuity in surface area (arrow). **(C)** Example of SR250 group - grade 3, demonstrates vertical fissure in superficial layer (arrow). **(D)** Example of SR500 group – grade 1, with slight discontinuity in surface area (arrow). **(E)** Example of SAL group - grade 4, with erosion reaching mid layer. **(F)** Example of SAL group - grade 5, with ulceration and fissure reaching the deep layer of articular cartilage (arrow), with areas of fibrocartilaginous repair (stars).

The histopathologic evaluation using the OARSI scoring system revealed that the SAL group had a higher grade classification than the other groups (4.25 ± 0.47), indicating a greater involvement of articular cartilage in those animals. The SrRan-treated groups (PROF250, SR250, and SR500) showed average grade classifications of 0.5 ± 0.22, 1 ± 0.7, and 0.75 ± 0.25, respectively, which were statistically similar to that of the CONTROL group (0.16 ± 0.16), with no significant difference between the two doses used (**Figures 6**, **7**). Therefore, a better histological profile of knee cartilage in the animals that received SrRan was observed, with a lower grade classification in the OARSI score compared to the SAL group.

## Discussion

The present study investigated the effects of SrRan, a medication that has been evaluated for possible inclusion in the class of disease-modifying OA drugs (DMOADs). To this end, we evaluated the effects of SrRan on pain behaviors in an experimental rat model of OA and described its effects on inflammation by determining cytokine levels in the synovial fluid and histopathological changes in affected joint sections.

The experiment was designed to mimic conditions with that would lead to the development of OA in the knee such as direct trauma, joint overload, improper alignment of the limb, quality of bone mass, and obesity (Allen and Golightly, 2015). Thus, the drug would have a preventive indication for administration before degenerative articular changes arise, which was assessed in the PROF250 group. In addition, we also assessed its effectiveness as a treatment in groups SR250 and SR500.

An improvement was observed in the joint incapacity following prophylactic use at the doses tested, and in the treatment of already established OA, although this effect lasted only up to D28. A study performed using a different rat model of OA induced with zymosan, a potent inducer of cyclooxygenase (COX)-2 expression, used doses ranging from 30 to 300 mg/kg/day for a shorter period (Nunes et al., 2015) and yielded similar results to those of this study. An experiment using much lower doses (25 and 50 mg/kg/day) and a method of OA induction similar to that used in the current study showed no reduction of joint incapacitation in animals treated prophylactically with SrRan or in those treated after the establishment of OA (Rodrigues et al., 2017). Therefore, the present study was the first to identify the beneficial effects of the preventive use of SrRan on joint activity.

SrRan (0.5, 5, and 50 mg/kg/day) also exhibited antinociceptive effects on the temporomandibular joint of rats in a zymosan-induced model of OA by reducing hypernociception, assessed using the Von Frey test. This phenomenon was verified, for the first time, in the present study at doses of 500 mg/kg/day, probably because the magnitude of OA induced in the knee was different from that induced in the temporomandibular joint (Alves et al., 2017). This was also verified at 300 mg/kg/day but not at 30 mg/kg/day in zymosan-induced knee OA (Nunes et al., 2015).

The benefit of the prophylactic use of SrRan was also verified by analyzing its effects on hyperalgesia induced by increasing the pressure on the rat’s paw, based on the fact that inflammation lowers the threshold of pain reactions. SrRan showed similar effects in other studies; however, these studies treated already established OA models and did not investigate the preventive effects (Alves et al., 2017; Rodrigues et al., 2017).

The model of OA induced by intra-articular injection of MIA enables pain assessment at an early stage of OA (Fernihough et al., 2004). Although the rats already presented structural changes consistent with OA as early as day 7 after induction, it is likely that the reduction of hypernociception by SrRan did not correlate only with structural modification, but also with changes in subtle biochemical mechanisms that mediate pain. OA is an autoinflammatory disease caused by chondrocyte- and synoviocyte-mediated responses, and the serum and synovial levels of inflammatory cytokines are higher in patients with OA than in those without the condition (Goldring and Otero, 2011; Berenbaum, 2013). To clarify the possible mechanisms mediating in this phenomenon, we determined the inflammatory mediators involved.

The results of the determination of inflammatory mediators in the synovial fluid following treatment with SrRan at different doses demonstrated a significant reduction of IL-6, TNF-α, and IFN-γ. These cytokines are known to be involved in the inflammatory cascade in the progression of OA, whereas there was an increase in IL-10, an anti-inflammatory cytokine (Pelletier et al., 2001; Goldring and Otero, 2011; Berenbaum, 2013). On the contrary, there was no difference between the treatment and prophylactic doses in the reduction of pro-inflammatory substances. This result corroborates available data showing a reduction in synovial fluid levels of TNF-α and IL-1β in zymosan-induced OA rats treated with SrRan at a dose of 300 mg/kg/day (Nunes et al., 2015). A decrease in the expression of TNF-α without changes in leukocyte count and IL-1β levels was also observed in another pre-clinical study of periarticular tissue samples, with lower doses of SrRan (up to 50 mg/kg/day) (Alves et al., 2017). These results were not confirmed in a study with rats subjected to oophorectomy and treated with SrRan at doses of 300 and 625 mg/kg/day, with no reduction of TNF-α and MMPs, which are metal-dependent endopeptidases that remodel the extracellular matrix (Mierzwa et al., 2017).

The histopathological analysis of articular cartilage using the OARSI classification in animals treated with SrRan showed encouraging results, suggesting the possible efficacy of SrRan in the prophylaxis and treatment of OA at the doses tested. The hyaline cartilage is the most relevant joint tissue in the pathogenesis of OA (Xia et al., 2014). Therefore, a drug with the potential to protect this tissue has excellent relevance. Thus, the protective effect of SrRan on cartilage demonstrated in the present study might justify its use for this purpose.

The scarcity of published experimental studies on SrRan use in OA, especially prophylaxis, makes a comparative evaluation of the results in this study a challenge. In rats subjected to oophorectomy, SrRan at doses of 300 mg/kg/day effectively attenuated the progression of OA, improving the quality of cartilaginous matrix by directly stimulating the synthesis of proteoglycans Furthermore, the cellular viability in those animals was preserved, with lower OARSI scores and reduced expression of caspase-3, an enzyme associated with apoptosis. This effect was abrogated with daily doses of 625 mg/kg associated with mechanical vibration (Mierzwa et al., 2017).

The SrRan-induced attenuation of degeneration of the articular architecture was also demonstrated in another rat model of OA induced by meniscal injury, with higher doses of the drug (625 and 1,800 mg/kg). The reduction of apoptotic indices of the chondrocytes was confirmed using terminal deoxynucleotidyl transferase-dUTP-nick end labeling (TUNEL) assay. Using micro-computed tomography to evaluate bone mineral density, an improvement in the rates of abnormality in the microarchitecture of the joints was observed. Microspectroscopy revealed an increase in the mineral-to-collagen ratio with SrRan treatment. Moreover, an increase in joint elasticity was observed using nanoindentation testing, a dynamic test for determining hardness. Additionally, an increase in the expression of sex determining region Y - box 9), a transcription factor with fundamental importance in chondrogenesis. Thus, treatment with high doses of SrRan showed positive results in controlling the deterioration of articular cartilage and subchondral bone remodeling (Yu et al., 2013).

A reduction in the progression of joint structural changes was also demonstrated by SrRan 25, 50, or 75 mg/kg/day in an experimental model of dogs subjected to anterior cruciate ligament transection. Effects such as reduction of the depth and size of articular lesions, in addition to greater preservation of the articular collagen network, were observed at doses of 25, 50, or 75 mg/kg/day using histomorphometric analysis. The expression of osteochondral degradation proteases (such as MMPs and cathepsin K) and IL-1β was reduced, especially with larger doses of the drug and longer time periods (Pelletier et al., 2012).

In contrast, a recent study with pigs administered a therapeutic dose of 625 mg/kg SrRan did not reveal any protective effects against degeneration of the articular cartilage in OA, determined using the OARSI classification scoring (Chu et al., 2017). The treated group in this study showed results similar to the control, which only showed improvements in the profile of the subchondral bone (Chu et al., 2017).

The results of the effects of SrRan on pain behavior, the inflammatory process, and the histological progression of OA presented in the current study were impressive with both prophylactic and therapeutic administration. However, this study has some limitations that need to be highlighted. Many of the beneficial effects observed with SrRan treatment in this and other pre-clinical studies, were observed at high doses of the drug, in contrast to doses of up to 2 g/day typically used in humans (Reginster, 2014). It is also worth noting the difficulty in reproducing OA with similar changes and evolution observed in humans since the experimental models still fail in this correlation (Pelletier et al., 2012). The lack of standardized clinical and experimental research methods for determining the effects of drugs for potential DMOAD classification is also a challenge in this field (Reginster et al., 2013).

The administration of SrRan in an MIA-induced experimental model of knee OA indicated its beneficial effect on pain, particularly in the improvement of articular incapacitation. This medication also mitigated histological changes in the articular cartilage and reduced the inflammatory process with lower synovial levels of IL-6, TNF-α, and IFN-γ than those in the untreated groups. In the challenging search for drugs that modify the normal pathogenesis and progression history of OA, pre-clinical results including those of this present study, support the potential use of SrRan for this purpose, both prophylactically and as a therapeutic agent.

## Author Contributions

TR, JG, and MdSdSC conceived of the presented idea. TR, JG, MdSdSC, and AdOF planned and carried out the experiments. GS, HC, and RG contributed to the histopathological analysis. TR, AdOF, GS, JV, MdSdSC, and JG contributed to the interpretation of the results. All the authors provided critical feedback and helped shape the research, analysis and manuscript.

## Conflict of Interest Statement

The authors declare that the research was conducted in the absence of any commercial or financial relationships that could be construed as a potential conflict of interest.
